# Identification of α2-macroglobulin as a biomarker for type 2 diabetes in human serum

**DOI:** 10.3389/fendo.2025.1534490

**Published:** 2025-05-23

**Authors:** Mingjie Wang, Limei He, Yuandi Chang, Zhaoli Yan

**Affiliations:** ^1^ Department of Endocrinology, Affiliated Hospital of Inner Mongolia Medical University, Hohhot, China; ^2^ Department of Internal Medicine, The First Hospital of Inner Mongolia Prison Administration, Hohhot, Inner Mongolia, China

**Keywords:** biomarkers, mass spectrometry, obesity, proteomics, type 2 diabetes mellitus, risk assessment, twodimensional polyacrylamide gel electrophoresis, α2-Macroglobulin

## Abstract

**Objectives:**

This study aimed to determine potential serum biomarkers of type 2 diabetes (T2DM) through proteomic data analysis and protein association assessment.

**Methods:**

This study included 80 patients with obesity, 76 patients with newly diagnosed T2DM combined with obesity, and 73 healthy controls. Proteomics analysis was used to investigate changes in protein abundance in the serum across the three groups. Correlations were analyzed using logistic regression, Pearson’s correlation, and Spearman’s correlation. Group comparisons for non-normally distributed continuous or categorical variables were performed using the Mann-Whitney U test, Kruskal-Wallis test, χ^2^ test, or Fisher’s exact probability test, as appropriate. Logistic regression analysis was employed to identify independent predictors, and correlations were evaluated using Pearson or Spearman tests based on data distribution. Receiver operating characteristic (ROC) curve analysis was employed to determine the predictive value of the differential proteins for the diagnosis of obesity and T2DM.

**Results:**

In this study, two-dimensional gel electrophoresis was used to analyze three groups. Several proteins were differentially expressed, with α2-macroglobulin (α2-MG) showing significant up-regulation in the obesity and T2DM + obesity groups compared to the control group. ELISA verification showed higher α2-MG levels in the obesity (2.746±0.391 g/L) and T2DM + obesity (3.261±0.400 g/L) groups than in the control group (1.376±0.229 g/L) (P<0.05). For predicting obesity and T2DM combined with obesity, α2-MG (AUC=0.873 and 0.601 respectively) were significant predictors.

**Conclusion:**

Serum a2-MG levels are elevated in obese individuals and those with T2DM. It shows high sensitivity and specificity for predicting obesity and T2DM, suggesting its potential as a biomarker for T2DM diagnosis. However, further large-scale studies are needed to confirm its clinical utility.

## 1 Introduction

Diabetes mellitus (DM) is one of the most prevalent metabolic disorders worldwide, posing a significant challenge to global public health systems. Notably, the global prevalence of type 2 diabetes mellitus (T2DM) continues to escalate at an alarming rate, with an even more pressing concern being the increasing number of individuals with prediabetes. Epidemiological projections estimate that the number of prediabetes cases will exceed 470 million by 2030 (1). Importantly, 5–10% of individuals with prediabetes progress to T2DM annually (2, 3), and emerging evidence indicates that these individuals already exhibit early signs of complications, indicating that pathological metabolic dysregulation precedes clinical diagnosis (1). Current diagnostic frameworks predominantly rely on traditional biomarkers such as glycated hemoglobin (HbA1c) and plasma glucose, measured under fasting conditions or via oral glucose tolerance tests. However, the World Health Organization (WHO) explicitly advises against using HbA1c to define prediabetes (4), highlighting limitations in early identification and subsequent delays in intervention. Among numerous risk factors, obesity has been identified as a primary driver of prediabetes progressing to T2DM (5), though the underlying mechanisms remain incompletely understood. Addressing the substantial physiological and socioeconomic burdens imposed by DM necessitates urgent efforts to identify high-risk populations at the earliest stages.

Recent studies utilizing animal models have provided novel insights into the molecular mechanisms linking obesity to metabolic dysregulation. For instance, research on equine metabolic syndrome revealed significant upregulation of inflammatory proteins—including heat shock protein 90, α2-macroglobulin (α2-MG), and interleukin-1β—in the visceral adipose tissue of insulin-resistant horses, suggesting that chronic inflammation exacerbates insulin resistance (IR) and metabolic disturbances (6). Notably, clinical observations dating back to 1967 demonstrated elevated serum α2-MG levels in newly diagnosed patients with T2DM (7), suggesting that this protein may contribute to early tissue injury during diabetes pathogenesis and that its dynamic changes could serve as an indicator of metabolic imbalance. Although obesity is closely linked to chronic inflammation, not all obese individuals exhibit elevated inflammatory markers. For instance, some research shows that in cases of simple obesity, C-reactive protein (CRP) levels may not significantly increase, indicating individual differences in inflammation's role in obesity-related metabolic disorders (8). In contrast, α2-MG, which is closely related to obesity and IR, may exhibit more specific and sensitive expression changes in individuals with simple obesity and prediabetes, underscoring its potential value as a biomarker for early detection of metabolic imbalance.

Proteomics, the study of proteins in cells, tissues, and organs, including protein identification, quantification, intracellular localization, and protein-protein interactions, is increasingly used in medical research (9). By combining gel- and chromatography-based separation techniques with mass spectrometry (MS) analysis and bioinformatics, proteomics can address various medical and basic science challenges. This technology enables the precise and reliable identification and quantification of proteins, which is crucial for the identification, validation, and application of disease-related biomarkers in diagnosis, prediction, and treatment (10). Specifically, proteomics can transform T2DM biomarkers into effective tools for screening, diagnosis, and prognosis long before the onset of clinical symptoms (11).

Tandem mass spectrometry (MS/MS) is currently used in newborn screening to detect conditions such as phenylketonuria (via phenylalanine and tyrosine) (12) and medium-chain acyl-CoA dehydrogenase deficiency (via acylcarnitine and medium-chain fatty acids) (13). When applied in national screening programs, MS/MS has demonstrated that metabolite biomarkers exhibit sufficient sensitivity and specificity for clinical use while remaining economically efficient. Currently, the diagnosis of diabetes is based solely on fasting plasma glucose (FPG), 2-h blood glucose, or glycosylated HbA1c levels. Although these novel T2DM biomarkers are effective, they tend to underestimate the prevalence of T2DM and do not provide guidance for early prediction. Given the economic and physical impacts of diabetes, identifying high-risk individuals as early as possible is of paramount clinical importance. Moreover, there is no accurate method for assessing changes in β-cell function during the dynamic pathological transition from obesity to T2DM (14).

Therefore, this study aimed to analyze proteomic data using two-dimensional gel electrophoresis (2-DE) and electrospray-quadrupole time-of-flight MS/MS (ESI-Q-TOF-LC-MS/MS) to identify potential proteins that may serve as diagnostic or predictive biomarkers of T2DM in obese individuals.

## Materials and methods

### Ethical consideration

This proteomic analysis and validation study was approved by the Medical Ethics Committee of Inner Mongolia Medical University (approval number YKD 202402082). Written informed consent was obtained from all participants in accordance with the regulations of the Chinese authority on research ethics.

### Study participants

Individuals diagnosed with obesity and new-onset T2DM combined with obesity by the Physical Examination Center and Endocrinology Department of the Affiliated Hospital of Inner Mongolia Medical University, respectively, were enrolled in this study. The inclusion criteria were as follows: 1) males aged 40–55 years; 2) an initial fasting plasma glucose ≥7.0 mmol/L, meeting the diagnostic criteria for DM; 3) no DM symptoms or hypoglycemic, antihypertensive, or lipid-lowering drug usage within the previous six months; 4) complete physical examination data; and 5) a body mass index (BMI) >30 kg/m^2^, meeting the criteria for obesity. Obesity was defined according to WHO criteria as a BMI≥30 kg/m². The exclusion criteria were as follows: 1) overweight due to other causes, including Cushing's syndrome, hypothyroidism, hereditary diseases, or pregnancy; 2) prior DM diagnosis; 3) use of hypoglycemic, antihypertensive, and lipid-regulating drugs; 4) use of immunomodulating drugs within the previous three months; 5) a history of surgery and trauma; 6) presence of infectious diseases, tumors, blood diseases, severe liver or kidney dysfunction, and autoimmune diseases; 7) serum CRP >10 mg/L, which may suggest underlying infections, tumors, and other diseases. Healthy volunteers were recruited from the Physical Examination Center of the Affiliated Hospital of Inner Mongolia Medical University.

### Clinical and laboratory data collection

The demographic and clinical characteristics of participants, including age, gender, height, and weight, were collected. BMI was calculated as: weight (kg)/height (m)². Biochemical parameters measured were as follows: triglycerides, high-density lipoprotein cholesterol, low-density lipoprotein cholesterol, alanine aminotransferase, alanine aminotransferase, gamma-glutamyl transpeptidase, glutamic dehydrogenase, glutamic acid dehydrogenase, total bilirubin, direct bilirubin, and FPG.

### Blood sample collection and preparation

Blood samples were collected from all participants using conventional venous blood sampling. An additional 3 mL sample was collected in BD Vacutainer serum separation tubes for proteomic experiments and enzyme-linked immunosorbent assay (ELISA). The tubes were allowed to clot at room temperature for 30 min, before undergoing centrifugation at 3000 × g for 10 min in a swinging bucket centrifuge. The separated serum was transferred into 1.5 mL tubes in 500 µL aliquots and stored at -70°C until further analysis.

### Two-dimensional gel electrophoresis and image analysis

Sample buffer containing 7 M urea, 2 M thiourea, 4.5% CHAPS, 100 mM DTE, and 40 mM Tris (pH 8.8) was added to immobilized pH strips (pH 3–10, ReadyStrip IPG; Bio-Rad Laboratories, USA). Isoelectrofocusing was performed by gradually increasing the voltage to 10,000 V. The second dimension of 2-DE was analyzed on 12% linear gradient polyacrylamide gels (20 cm × 25 cm × 1.5 mm) at 40 mA and then at 150 mA for approximately 6 h. The gels were fixed overnight, stained with silver, destained with H_2_O, scanned using a GS710 densitometer (Bio-Rad, Richmond, CA, USA), and converted to electronic files. Image analysis was conducted using Image Master Platinum 5.0 (Amersham Biosciences, Amersham, UK).

### Mass spectrometry

Proteomic analysis was performed using ESI-Q-TOF-LC/MS. Serum samples were separated on a C18 nanobore column (150 mm × 0.1 mm, 3 µm pore size; Beijing Zhengdan International Science and Technology Co., Ltd., Beijing, China). The mobile phase A for LC separation was 0.1% formic acid, while the mobile phase B was 0.1% formic acid in acetonitrile. The chromatographic gradient was designed to increase linearly from 0% B to 40% B in 33 min, 40% B to 80% B in 2 min, and 5% B in 5 min. The flow rate was maintained at 400 nL/min. Mass spectra were obtained by data-dependent acquisition with a full mass scan (100–2500 Da) at a capillary voltage of 1,500 V in a positive ion detection mode with a drying nitrogen gas flow rate of 4 L/min at 150°C.

### Database search

The Mascot algorithm (Matrix Science, Boston, MA, USA) was utilized to identify peptide sequences in the protein sequence database. The database search parameters were as follows: *Homo sapiens*; carbamidomethyl (C) variable modification; oxidation; peptide tolerance, 0.05 Da; MS/MS tolerance, 0.1 Da; and significance threshold, P<0.05.

### Enzyme-linked immunosorbent assay

Serum levels of identified candidate proteins were measured using ELISA (Quanzhou Ruixin Biotech and Nanjing Jiancheng Bioengineering Institute, China) in accordance with the manufacturer’s instructions. Specifically, the ELISA for serum α2-MG included sample preparation, reagent preparation, experimental procedures, and result calculation. For sample preparation, serum α2-MG was diluted 400 times by mixing 5 μL of sample with 95 μL of sample diluent (1:20 dilution), then transferring 15 μL to 285 μL of sample diluent to achieve a final 1:400 dilution. Reagent preparation involved reconstituting the α2-MG standard lyophilized powder with standard diluent to 150 μL, then using 5 centrifuge tubes (marked S5 to S1) for serial dilution. Each tube received 250 μL of standard diluent, and the standard original solution (250 μL) was added to S5, mixed, then sequentially transferred to the next tube down to S1. The biotin-labeled antibody working solution was diluted 1:100 with its diluent. The experimental steps were as follows: Reagents were balanced to room temperature (18–25°C) for at least 30 min. For sampling, standard and test sample wells were set up, each receiving 100 μL of standard or sample, mixed, covered, and incubated at 37°C for 2 h. After discarding the liquid and drying, 100 μL of biotin-labeled antibody working solution was added to each well, covered, and incubated at 37°C for 1 h. After discarding and drying, the plates were washed 3 times (2 min each, 200 μL per well). Then, 100 μL of horseradish peroxidase-labeled streptavidin working solution was added to each well, covered, and incubated at 37°C for 1 h. Following another wash (5 times), 90 μL of substrate solution was added to each well, and then incubated at 37°C in the dark for 15–30 min. The reaction was stopped by adding 50 μL of stop solution to each well. The optical density (OD) at 450 nm was measured within 5 min using a microplate reader. The concentration of α2-MG in the samples was determined by plotting the OD values against the standard curve and multiplying by the dilution factor for diluted samples.

### Statistical analysis

Statistical analyses were performed using SPSS version 21 (IBM SPSS Inc., Armonk, NY, USA). Measurement data were tested for normality using the Shapiro-Wilk test. Normally distributed data are described using mean ± standard deviation (X±S) and compared between groups via one-way analysis of variance (ANOVA). Non-normally distributed continuous variables are expressed using P50 (P25, P75). Categorical data are presented as the number of cases and percentages (%). Correlations were analyzed using logistic regression, Pearson’s correlation, and Spearman’s correlation. Group comparisons for non-normally distributed continuous or categorical variables were performed using the Mann-Whitney U test, Kruskal-Wallis test, χ² test, or Fisher’s exact probability test, as appropriate. Logistic regression analysis was employed to identify independent predictors, and correlations were evaluated using Pearson or Spearman tests based on data distribution. Receiver operating characteristic (ROC) curve analysis was employed to determine the predictive value of the differential proteins for the diagnosis of obesity and T2DM. P-values <0.05 were considered statistically significant.

## Results

### Participant characteristics

The participant characteristics are summarized in **Table 1**. A total of 229 individuals were enrolled in this study, comprising 73 healthy volunteers, 80 patients with simple obesity, and 76 patients with newly diagnosed T2DM and obesity. No significant differences in age were observed among the three groups. The healthy control group exhibited significantly lower BMI values than both the obesity and newly diagnosed T2DM+obesity groups (P<0.05), while no significant difference was observed between the latter two groups. Regarding glycemic profiles, FPG levels in the newly diagnosed T2DM+obesity group were significantly higher than those in both the healthy control and obesity groups (P<0.05), while no significant difference was detected between the obesity and healthy control groups. In contrast, fasting serum insulin levels in the newly diagnosed T2DM+obesity group were markedly lower than those in the healthy control and obesity groups (P<0.05). IR, assessed using the homeostatic model assessment of IR (HOMA-IR), was significantly elevated in the newly diagnosed T2DM+obesity group compared to the other two groups (P<0.05), indicating the most severe IR in this cohort.

**Table 1:** Demographic and clinical characteristics of the study participants.

Characteristic	Control group (n=73)	Obesity group (n=80)	T2DM+obesity group (n=76)	Z/F	*P*
Age	39.986 ± 7.245	38.625 ± 7.036	40.855 ± 6.711	2.018	0.135
BMI^a^ (kg/m^2^)	22.392 ± 1.737	27.147 ± 2.220^e^	27.834 ± 3.137^e^	109.801	0.000
GLU^b^ (mmol/L)	4.920 ± 0.380	5.034 ± 0.391	8.259 ± 2.099^ef^	175.016	0.000
FINS^c^ (mIU/L)	3.901 ± 1.388	3.975 ± 1.342	2.531 ± 1.109^ef^	34.592	0.000
HOMA-IR^d^	0.879 ± 0.311	0.886 ± 0.304	1.067 ± 0.530^ef^	5.116	0.007

Values are presented as mean ± standard deviation.

^a^BMI, body mass index; ^b^GLU, glucose; ^c^FINS, fasting serum insulin; ^d^HOMA-IR, homeostatic model assessment of insulin resistance. Compared with the control group ^e^ *P*<0.05; Compared with the obesity group ^f^ *P*<0.05.

### 2-DE and image comparison

**Figure 1:**
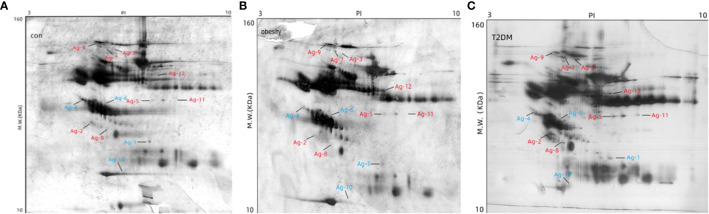
Determination of differential abundance of proteins between study populations. Two-dimensional gel electrophoresis of the extracted proteins was used to compare the 12 differentially expressed proteins among the three groups: **(A)** control, **(B)** obesity, and **(C)** T2DM+obesity. MW, molecular weight; con, control group; obesity, obesity only group; T2DM+obesity, type 2 diabetes with obesity group.

Changes in serum protein were assessed using 2-DE in 10 healthy controls, 10 individuals with obesity alone, and 10 patients with new-onset T2DM+obesity. Between the three groups, a total of 12 significant spots on the 2D gels were observed (indicating protein expression), of which 8 were over-expressed and 4 were under-expressed. These were identified based on a minimum 2.5-fold difference in expression compared with the control group (**Figure 1**).

### Identification of differentially expressed proteins

Gel scan analyses revealed a mean of 113 ± 33, 126 ± 39, and 123 ± 27 protein spots in the control, obesity, and T2DM+obesity groups, respectively. Further analysis confirmed 12 protein spots expressed at various abundances among the control, obesity, and T2DM+obesity groups (ratio >2.0, P<0.01). Upon subjecting the 12 spots to LC-MS/MS for protein identification (**Table 2**), a number of proteins were identified including complement C4-A, clusterin, α2-MG, zinc- α2-glycoprotein (ZAG), complement C3 (C3), apolipoprotein L1, α1-antitrypsin (AAT), and complement factor B. Among these, C3 (protein accession number gi|718) and α2-MG (protein accession number gi|177870) were found to be under-expressed in the control group compared with the other two groups, while AAT (protein accession number gi|177829) and ZAG (protein accession number gi|220151) were over-expressed in the control group compared with the obesity and T2DM+obesity groups. Notably, the elevated complement C3 and α2-MG levels in the obesity and T2DM+obesity groups corresponded with elevated insulin and glucose levels. Additionally, the increase in zinc-α2-glycoprotein levels in obesity and T2DM+obesity groups were consistent with the IR among these individuals compared to the insulin-sensitive healthy controls.

**Table 2:** Differentially expressed proteins identified using 2-DE image analysis.

Group no.	Protein accession number	Protein name	Mr^a^	pI^b^	Score
Ag-1	gi|476007827	Complement C4-A	194261	6.65	1533
Ag-2,8	gi|2247796463	Clusterin	53031	5.89	3206
Ag-3	gi|177870	α-2-Macroglobulin	164613	6.03	2312
Ag-4	gi|220151	Zinc-α-2-glycoprotein	34465	5.71	5376
Ag-5,11	gi|718	Complement C3	188569	6.02	12062
Ag-6,10	gi|13374347	Apolipoprotein L1	44004	5.60	3877
Ag-7	gi|718	Complement C3	188569	6.02	6105
Ag-9	gi| 1703025	α1-Antitrypsin	46878	5.37	4851
Ag-12	gi|297569	Complement factor B	86847	6.67	5228

^a^Mr, molecular mass; ^b^pI, isoelectric point.

### Quantification of differentially expressed serum proteins

To validate the identified candidate proteins from the proteomic analysis, serum AAT, C3, ZAG, and α2-MG levels were measured using ELISA (**Table 3**). No significant associations between protein levels and age or sex were observed in any group. Notably, C3 levels were significantly higher in the T2DM+obesity group (1.565 ± 0.296 g/L) than in the control group (1.076 ± 0.303 g/L). The same goes for the obesity group (1.201 ± 0.302 g/L), where C3 levels were higher than those of the control group but significantly lower than those in the T2DM+obesity group (1.565 ± 0.296 g/L) (P<0.05). The α2-MG levels followed a similar expression pattern as that of C3, with significantly higher α2-MG levels in the obesity (2.746 ± 0.391 g/L) and T2DM+obesity (3.261 ± 0.400 g/L) groups than in the control group (1.376 ± 0.229 g/L) (P<0.05). Conversely, serum AAT levels were significantly lower in the T2DM+obesity group (2.539 ± 0.516 g/dL) than in the obesity (2.753 ± 0.454 g/dL) and control (3.086 ± 0.596 g/dL) groups (P<0.05). Similarly, serum AAT and ZAG levels were lower in the obesity group than in the control group (P<0.05).

**Table 3:** Group comparison of AAT, C3, ZAG, and α2-MG levels.

Indicator	Control group (n=73)	Obesity group (n=73)	T2DM+obesity group (n=73)	*F*	*P*
AAT (g/dL)	3.086 ± 0.596	2.753. ± 0.454^a^	2.539 ± 0.516^ab^	20.651	0.000
C3 (g/L)	1.076 ± 0.303	1.201 ± 0.302^a^	1.565 ± 0.296^ab^	53.876	0.000
ZAG (μg/ml)	57.578 ± 25.767	47.968 ± 31.916^a^	44.303 ± 23.630^a^	4.636	0.011
α2-MG (g/L)	1.376 ± 0.229	2.746 ± 0.391^a^	3.261 ± 0.400^ab^	571.037	0.000

Compared with the control group, ^a^ *P*<0.05; Compared with the obesity group, ^b^ *P*<0.05.

### Multivariate multifactor logistic regression analysis

To further examine the correlation between AAT, C3, ZAG, and α2-MG across the three groups, we performed multivariate logistic regression using the group as the dependent variable and AAT, C3, ZAG, and α2-MG levels as the independent variables, with the control group as the reference (**Table 4**). After adjusting for age, HOMA-IR, and other relevant indicators, increased levels of C3 (OR=3.580, P<0.01, 95% confidence interval [CI]:1.460–8.780) and α2-MG (OR=11.536, P<0.01, 95%CI:5.311–25.057) in the obesity group were found to be significant factors associated with obesity development when compared to the control group. Additionally, in the T2DM+obesity group, reduced AAT (OR=0.997, P<0.05, 95%CI:0.996–0.998) and increased C3 (OR=7.202, P<0.01, 95%CI:2.643–19.626) and α2-MG (OR=16.801, P<0.01, 95%CI: 7.401–38.13) levels were found to influence T2DM development.

**Table 4:** Multivariate multifactorial logistic regression analysis.

Group	Indicator	β^a^	Standard error	Wald^b^	OR^c^	95%CI^d^		*P*
						Lower limit	Upper limit	
Obesity group	Intercept	-0.974	1.727	0.318				0.573
	AAT	-0.001	0.000	8.775	0.999	0.998	1.000	0.003
	C3	1.275	0.458	7.767	3.580	1.460	8.780	0.005
	ZAG	-0.016	0.008	3.548	0.984	0.968	1.001	0.060
	α2-MG	2.445	0.396	38.180	11.536	5.311	25.057	0.000
	HOMA-IR	-1.042	0.638	2.665	0.353	0.101	1.232	0.103
T2DM+obesity group	Intercept	-1.337	1.814	0.543				0.461
	AAT	-0.003	0.001	24.992	0.997	0.996	0.998	0.000
	C3	1.974	0.511	14.903	7.202	2.643	19.626	0.000
	ZAG	-0.017	0.009	3.640	0.983	0.965	1.000	0.056
	α2-MG	2.821	0.418	45.502	16.801	7.401	38.139	0.000
	HOMA-IR	0.615	0.617	0.996	1.850	0.553	6.193	0.318

^a^β, coefficient estimate; ^b^Wald, chi-squared value; ^c^OR, order of magnitude, indicating the number of units added to the experimental variable; ^d^CI, confidence interval.

### Sensitivity and specificity analysis of target proteins

**Figure 2:**
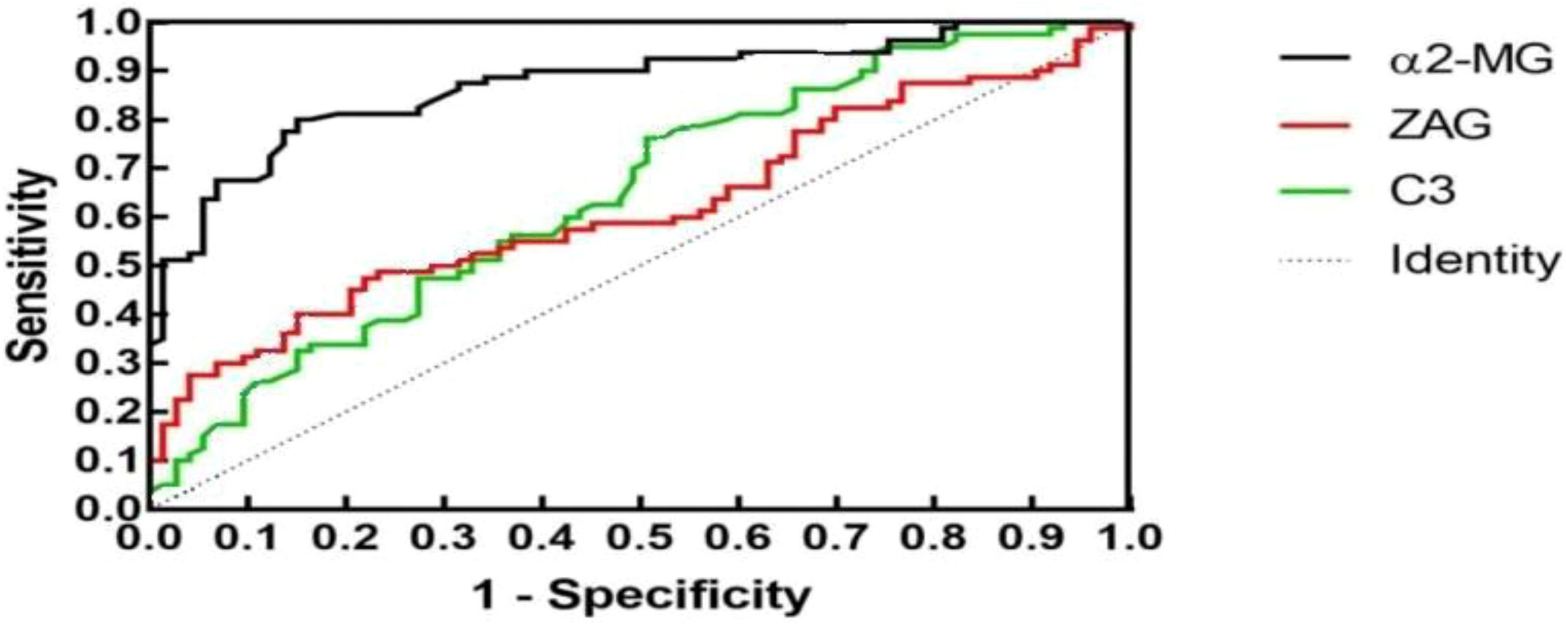
ROC plots for the control and obesity groups.

**Figure 3:**
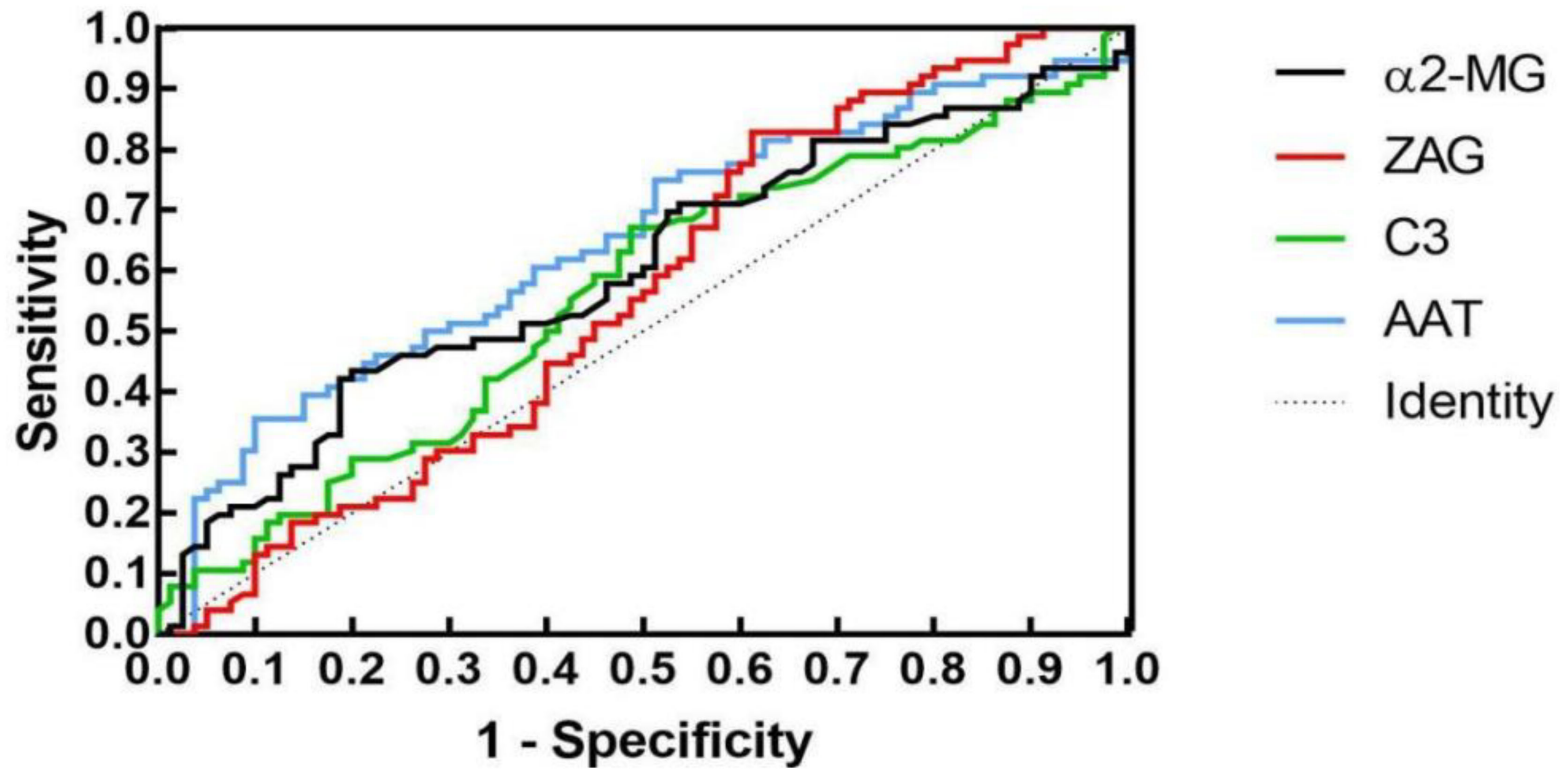
ROC plots for the control and T2DM+obesity groups.

To assess whether C3, ZAG and α2-MG could serve as accurate diagnostic biomarkers of T2DM, we performed ROC curve analysis to evaluate their positive and negative predictive values for obesity and T2DM+obesity development, with the prediction thresholds determined using the maximum Youden's index cut-off point (**Figures 2**, **3**). ROC analysis comparing the control and obesity groups revealed that C3, ZAG and α2-MG were all significant predictors of obesity (P<0.05; **5A**), with α2-MG exhibiting the highest sensitivity (AUC=0.873), followed by C3 (AUC=0.763) and ZAG (AUC=0.620). Moreover, when comparing the control group with the T2DM+obesity group, AAT (AUC=0.646) and α2-MG (AUC=0.601) emerged as significant predictors of T2DM+obesity (P<0.05; **Table 6A**). Notably, C3 and ZAG, which proved to be positive diagnostic indicators of obesity alone, were not sensitive predictive indicators of obesity when coupled with T2DM. These findings suggest that α2-MG might be a potential biomarker for predicting T2DM in obese individuals due to its enhanced sensitivity and specificity compared with other potential target proteins. **Tables 5B** and **6B** illustrate the sensitivity, specificity, likelihood ratios, and predictive values for each protein according to obesity and T2DM+obesity development, respectively (P<0.05).

**Table 5A:** ROC analysis for each indicator in the control and obesity groups.

Indicator	AUC	SE	*P*	95%CI		Cut-off
				Lower limit	Upper limit	
C3	0.646	0.044	0.002	0.560	0.733	0.995
ZAG	0.620	0.045	0.011	0.531	0.709	47.485
α2-MG	0.873	0.029	0.000	0.817	0.929	2.230

**Table 5B:** Control and obesity group diagnostic indicators.

Indicator	Sensitivity	Specificity	Youden’s index	PPV^a^	NPV^b^	+LR^c^	−LR^d^
C3	0.763	0.493	0.256	0.622	0.655	1.504	0.482
ZAG	0.537	0.630	0.167	0.614	0.554	1.453	0.734
α2-MG	0.775	0.863	0.638	0.861	0.778	5.658	0.261

^a^PPV, positive predictive value; ^b^NPV, negative predictive value; ^c^+LR, positive likelihood ratio; ^d^-LR, negative likelihood ratio; AUC, area under the curve; SE, standard error; CI, confidence interval.

**Table 6A:** ROC analysis for each indicator in the control and T2DM+obesity groups.

Indicator	AUC	SE	*P*	95%CI		Cut-off
				Lower limit	Upper limit	
AAT	0.646	0.044	0.002	0.559	0.733	2.189
C3	0.559	0.046	0.202	0.468	0.650	1.215
ZAG	0.559	0.046	0.205	0.468	0.650	41.940
α2-MG	0.601	0.046	0.029	0.512	0.691	3.340

**Table 6B:** Control and T2DM+obesity group diagnostic indicators.

Indicator	Sensitivity	Specificity	Youden’s index	PPV^a^	NPV^b^	+LR^c^	−LR^d^
AAT	0.355	0.900	0.255	0.771	0.595	3.553	0.716
C3	0.671	0.488	0.184	0.567	0.621	1.377	0.642
ZAG	0.568	0.600	0.166	0.518	0.548	1.132	0.868
α2-MG	0.421	0.812	0.234	0.681	0.596	2.246	0.713

^a^PPV, positive predictive value; ^b^NPV, negative predictive value; ^c^+LR, positive likelihood ratio; ^d^-LR, negative likelihood ratio; AUC, area under the curve; SE, standard error; CI, confidence interval.

## Discussion

This study examined changes in the serum proteome of healthy individuals and clinically obese individuals using 2-DE and LC-MS/MS to determine diagnostic or predictive markers for the progression of obesity to T2DM. Among the 12 identified proteins, AAT, C3, ZAG, and α2-MG exhibited significant differences between groups. Compared to the healthy controls, patients with obesity and newly diagnosed patients with T2DM with obesity exhibited an increasing trend in C3 and α2-MG levels and a decreasing trend in AAT and ZAG levels. In middle-aged and young males, serum α2-MG exhibited high sensitivity and specificity for predicting obesity and T2DM. While reduced AAT levels exhibited high specificity for predicting T2DM, serum C3 and ZAG levels had limited predictive value for obesity and T2DM. The current literature on these proteins supports our findings.

### Alpha-1 antitrypsin

AAT is a protease inhibitor primarily synthesized by hepatocytes that mainly functions to inhibit neutrophil elastase, a serine protease released by neutrophils, thereby protecting tissues from protease-induced damage. Consequently, it is also known as α1-proteinase inhibitor (15). Beyond its protease-inhibitory activity, AAT neutralizes the effects of inflammatory mediators, demonstrating anti-inflammatory and immunomodulatory effects (16), as well as anti-apoptotic (17) and cytoprotective (15) functions.

In the present study, serum AAT levels were lower in the newly diagnosed T2DM group than in the other two groups, with the obesity group exhibiting lower levels than those in the control group. When AAT levels were <2.189 g/dL, the sensitivity for predicting T2DM development in patients with obesity was low, whereas the specificity was high (90.0%), indicating a low false-positive rate.

Swiatkowska-Stodulska et al. (18) reported that obese individuals with metabolic syndrome exhibited higher serum AAT levels than those without metabolic syndrome. However, when considering obesity alone, AAT levels did not significantly differ from controls. Another study found that elevated plasma AAT levels could predict the incidence of cardiovascular diseases but not diabetes. However, this study did not record BMI and thus did not investigate the relationship between plasma AAT levels and diabetes incidence in patients with obesity (19). These findings suggest that AAT levels can serve as biomarkers for predicting T2DM development in overweight and obese populations.

Research indicates that AAT levels are reduced in metabolic syndrome-related diseases such as T2DM, ischemic stroke, and non-alcoholic fatty liver disease (20). This may be attributed to the action of circulating neutrophil elastase, with high plasma concentrations observed in T2DM and non-alcoholic fatty liver disease. The imbalance between neutrophil elastase and AAT levels has been shown to contribute to the development of obesity, related inflammation, IR, and hepatic steatosis (21). Kim et al. (22) also observed reduced serum AAT levels in prediabetic and diabetic patients with abdominal obesity. Emerging evidence suggests that low-grade systemic inflammation can stimulate AAT secretion; however, this balance is disrupted when diabetes occurs, leading to reduced serum AAT levels and impaired AAT activity. These findings suggest that AAT expression, secretion, and activity vary depending on the degree of metabolic disturbance. Although the underlying mechanisms are not fully understood, AAT may serve as a novel biomarker of obesity and T2DM.

### Complement protein C3

C3, a common inflammatory marker (23), is the most abundant complement component in serum and plays a crucial role in the activation of the three pathways of the complement system, which is involved in insulin secretion promotion, fat metabolism regulation, and energy storage. Activation of C3 has been linked to various metabolic abnormalities. C3 degradation products are similar to acylation-stimulating protein (ASP), which enhances glucose uptake and lipid synthesis in adipocytes, thereby promoting fat accumulation. Additionally, C3 can induce M1 macrophage aggregation in adipose tissues and apoptosis induction in pancreatic β-cells (24).

A seven-year prospective cohort study demonstrated that C3 levels were independently associated with the incidence of T2DM (25). Changes in C3 levels are closely related to changes in IR in muscle, liver, and adipose cells. Although C3 itself may not actively promote IR development, it serves as a marker of adipocyte dysfunction. Adipocytes produce C3 and convert it into C3a, with the final product, C3a-desArg, also known as ASP. Similar to insulin, ASP promotes glucose and lipid storage in adipocytes. Thus, serum C3 levels positively correlate with IR and glucose tolerance. Baseline C3 levels can predict T2DM onset to some extent (25). Multiple studies have reported higher C3 levels in patients with T2DM than in healthy individuals (26–28).

In the present study, C3 levels were higher in the newly diagnosed T2DM group than in the other two groups, and higher in the obesity group than in the control group. When a C3 cut-off value of >1.215 g/L was applied, the sensitivity for predicting T2DM in obese individuals was 67.1%, while the specificity was 48.8%. Since the sensitivity and specificity were moderate, we inferred that C3 could not be used as a predictive or exclusionary indicator of T2DM. However, this finding suggested that C3 increased with the progression of IR and chronic inflammation in obesity and T2DM. Thus, obesity and hyperglycemia in humans may contribute to a state of low-grade persistent chronic inflammation, which not only directly activates the complement system, but also causes abnormal complement regulation. This, in turn, indirectly activates the complement system, further mediates inflammatory responses, and causes more severe abnormalities in lipid metabolism. High C3 levels can also promote pancreatic β-cell apoptosis, leading to reduced insulin secretion and T2DM exacerbation. Therefore, serum C3 levels have significant implications in T2DM development and progression.

### Zinc-α2-glycoprotein

ZAG, a major class I histocompatibility complex molecule, has been shown to promote lipid metabolism, glucose utilization, and insulin sensitivity regulation. ZAG possesses 2 high-affinity binding sites and 15 low-affinity binding sites for zinc, which influence its interactions with fatty acids and β-adrenergic receptors (29). ZAG has also been recognized as a lipid mobilizing adipokine (30). In humans, serum ZAG levels have been shown to be positively correlated with serum triglyceride and adipocyte fatty acid-binding protein levels, and negatively correlated with high-density lipoprotein cholesterol levels (31). ZAG can increase body temperature, promote weight loss, and reduce adipose tissue by enhancing uncoupling protein expression, leading to increased energy expenditure (32). However, the precise mechanisms underlying its influence on lipid metabolism remain unclear and require further investigation.

In the present study, ZAG levels were lower in the obesity and newly diagnosed T2DM+obesity groups than in the control group. ROC analysis revealed that a ZAG cut-off value of <47.485 μg/mL exhibited a sensitivity of 53.7% and specificity of 63.0% for predicting obesity, whereas a ZAG cut-off value of <41.940 μg/mL exhibited a sensitivity of 56.8% and specificity of 60.0% for predicting T2DM in patients with obesity. Since both specificity and sensitivity percentages were moderate, it was inferred that ZAG is not an ideal predictive marker for obesity and T2DM.

Research on the effects of ZAG on glucose metabolism is limited. In mice, the intravenous injection of ZAG has been shown to reduce FPG levels and improve glucose tolerance without altering plasma insulin levels 30 min after oral glucose administration (33). Russell et al. (34) found that ZAG can reduce circulating glucose levels and increase basal glucose uptake in adipocytes through the overexpression of glucose transporter 4 via β1-adrenergic receptor activation. However, plasma glucose levels and plasma ZAG levels were not directly correlated. Wang et al. (35) reported that serum ZAG levels were reduced in patients with metabolic syndrome and central obesity, and that decreased serum ZAG levels were associated with an increased risk of metabolic syndrome. Hence, serum ZAG levels, particularly the serum ZAG/fat mass ratio, may be candidate diagnostic markers for metabolic syndrome. ZAG may promote glucose utilization, storage, and excretion, with β-adrenergic receptors playing an important role in ZAG-regulated glucose metabolism. However, the specific mechanisms underlying these effects require further investigation.

Many researchers believe that ZAG is associated with the development of diabetic nephropathy. In an Egypt-based case-control observational study, serum ZAG levels in patients with T2DM were significantly higher than those in the control group, indicating that its potential as a useful biomarker for early diagnosis (36). Similarly, Sonkar et al. (37) proposed ZAG as an early biomarker for diabetic nephropathy, noting a reduction with disease progression. Since ZAG exhibits anti-inflammatory properties, its depletion can exacerbate disease. Severo et al. (38) reported that ZAG levels were negatively correlated with BMI and weight and identified zinc as an important regulator of ZAG homeostasis in the body, with changes in zinc metabolism during obesity impairing ZAG function. These results suggest that ZAG is involved in lipid and glucose metabolism, insulin sensitivity regulation, and inflammatory responses. Therefore, ZAG may be a useful biomarker for the early diagnosis of diabetic nephropathy and warrants further longitudinal prospective studies to explore its potential clinical utility.

### Alpha-2-macroglobulin

α2-MG, a large molecular weight glycoprotein primarily found in human plasma, is locally synthesized in the liver by macrophages. Its synthesis and activation depend on the induction of various acute-phase proteinases, including cytokines. These acute-phase proteinases activate transcription factors, such as nuclear factor kappa B and CCAAT/enhancer binding protein beta/delta, stimulating the liver macrophages to produce and secrete α2-MG (39). α2-MG can modulate the activity of cytokines, hormones, growth factors, and other proteins (40). As a broad-spectrum protease inhibitor, α2-MG can clear both endogenous and exogenous proteases. Therefore, in diabetes, the upregulation of acute-phase proteins enhances α2-MG synthesis.

In the present study, α2-MG levels exceeding 2.230 g/L had a sensitivity of 77.5% and specificity of 86.3% for predicting obesity. ROC curve analysis indicated that α2-MG exhibited good predictive value for obesity, perhaps due to the fact that during the prediabetic stage of IR in obesity, the body urgently secretes protease inhibitors, including α2-MG. α2-MG exerts multifaceted effects on insulin signaling and improves IR by binding to low-density lipoprotein receptor-related protein-1 (LRP1). On one hand, the activation of LRP1 by α2-MG initiates the intracellular phosphoinositide 3-kinase/protein kinase B and mitogen-activated protein kinase/extracellular signal-regulated kinase signaling pathways. This activation promotes the expression of glucose transporter type 4 on the cell surface through Rab4-, Rab8A-, and Rab10-mediated recycling pathways, thereby enhancing insulin-induced glucose uptake efficiency (41, 42). On the other hand, α2-MG can block the binding of aggregated low-density lipoprotein to LRP1, inhibiting its internalization and reducing the accumulation of cholesteryl ester within cells. This effect helps maintain insulin sensitivity in tissues such as cardiomyocytes and alleviates IR caused by abnormal lipid accumulation, thus potentially playing a therapeutic role in diabetes and its associated cardiovascular diseases. However, as α2-MG levels increase, the bioavailability of insulin in the body may decrease, leading to impaired glucose regulation and further exacerbating IR. This may be related to the binding of serum α2-MG to insulin or its impact on the internalization of insulin by target cells (43, 44). Notably, the specificity of serum α2-MG for diagnosing male T2DM was high, with a low misdiagnosis rate.

Takada et al. (45) demonstrated that serum α2-MG exhibited a specific expression pattern in DM and its complications. Patients with diabetic retinopathy showed elevated α2-MG levels compared to healthy controls, with a significant positive correlation to HbA1c, a glycemic control indicator. Notably, α2-MG expression closely correlated with the progression of diabetic nephropathy. In patients with albuminuria (<800 mg/day), α2-MG levels increased alongside urinary protein excretion, suggesting its potential as a dynamic biomarker for monitoring microvascular complications. Additionally, Caixeta et al. (46) reported that salivary α2-MG in patients with T2DM with poor glycemic control strongly correlated with HbA1c (r=0.838) but weakly with blood glucose (r=0.354), indicating its role in reflecting chronic hyperglycemic states rather than acute fluctuations—a characteristic consistent with HbA1c’s role in long-term glycemic monitoring. This supports the potential use of salivary α2-MG as a complementary indicator to HbA1c, offering clinical value for non-invasive diabetes monitoring. de Paula Silva et al. (47) further observed that α2-MG was significantly elevated in diabetic patients across all DM subtypes. These findings align with our conclusions, collectively underscoring α2-MG’s potential as a precursor biomarker for DM diagnosis. Importantly, α2-MG elevation may precede the onset of overt metabolic dysregulation. In our study, obese individuals with serum α2-MG levels significantly higher than healthy controls (P<0.001), even before reaching the diagnostic glycemic threshold for DM, suggest its utility as an early warning signal for the transition from IR to overt diabetes.

## Conclusions

This study employed 2-DE and LC/MS-Q-TOF technology to investigate the proteomics of human serum, linking the serum proteomes of healthy individuals, patients with obesity, and newly diagnosed patients with T2DM with obesity. We identified candidate proteins associated with obesity and newly diagnosed T2D, validated them using ELISA verification, and assessed their potential as novel biomarkers using logistic regression and ROC curve analyses. Among the 12 identified proteins, AAT, C3, ZAG, and α2-MG showed significant differences. Overall, our findings highlights the potential of these specific serum proteins as biomarkers for obesity and T2DM. However, further research with larger and more diverse populations is needed to validate these findings and explore their clinical utility. It is important to note that our study has some limitations. The sample size, although determined based on stringent inclusion and exclusion criteria to establish a well-defined and homogenous study population, may limit the representativeness of our sample to the broader population. Future studies should aim to recruit larger and more diverse cohorts to confirm our results and enhance their generalizability.

## Data Availability

The original contributions presented in the study are included in the article/**Supplementary Material**. Further inquiries can be directed to the corresponding author/s.
