# Supplementary material for: Identification of α2-macroglobulin as a biomarker for type 2 diabetes in human serum
Source: Front Endocrinol (Lausanne). 2025 May 23;16:1534490. doi: 10.3389/fendo.2025.1534490 (PMC12140998; doi:10.3389/fendo.2025.1534490)
Supplement: Supplementary file 1 [file DataSheet1.docx]

Supplementary Material

## Supplementary Figures


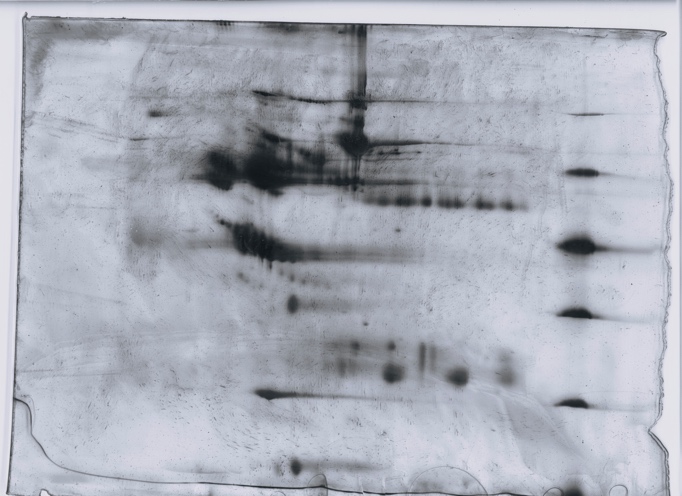


**Supplementary Figure 1.** Determination of protein abundance in the control group. Two-dimensional gel electrophoresis of the extracted proteins from healthy individuals was used to establish a baseline for protein expression levels. This figure serves as a reference for comparing protein expression changes in other study groups.


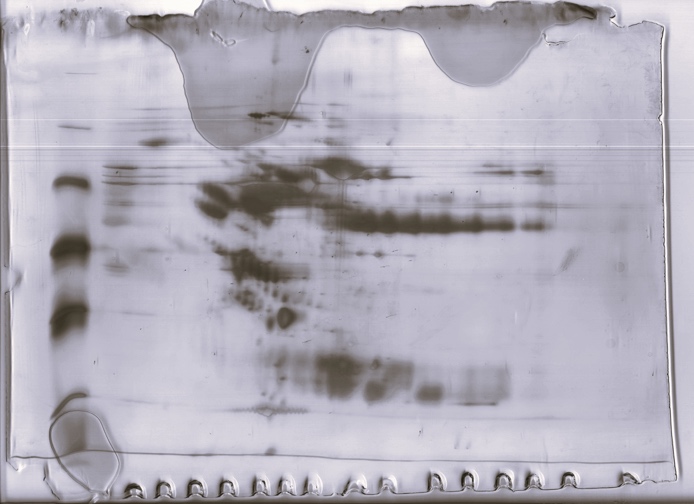

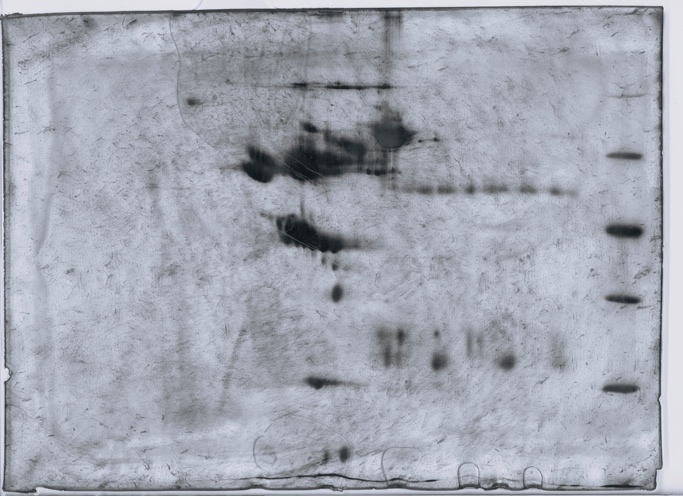


**Supplementary Figure 2.** Determination of differential abundance of proteins in the obesity group. Two-dimensional gel electrophoresis of the extracted proteins from individuals diagnosed with obesity was used to identify changes in protein expression levels compared to the control group. This figure highlights proteins that may be associated with obesity.


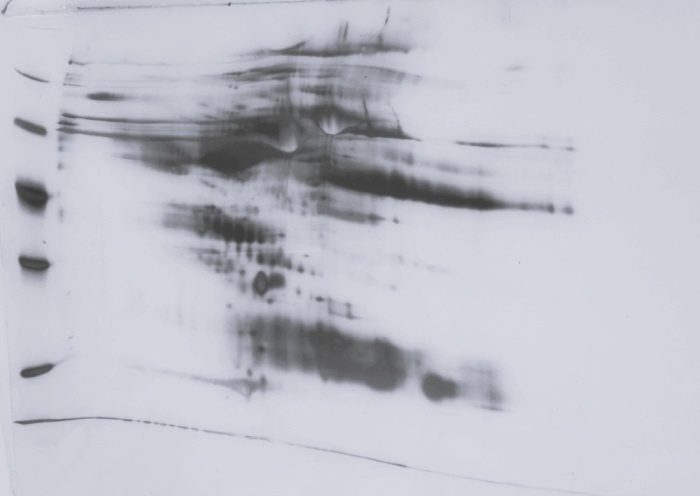

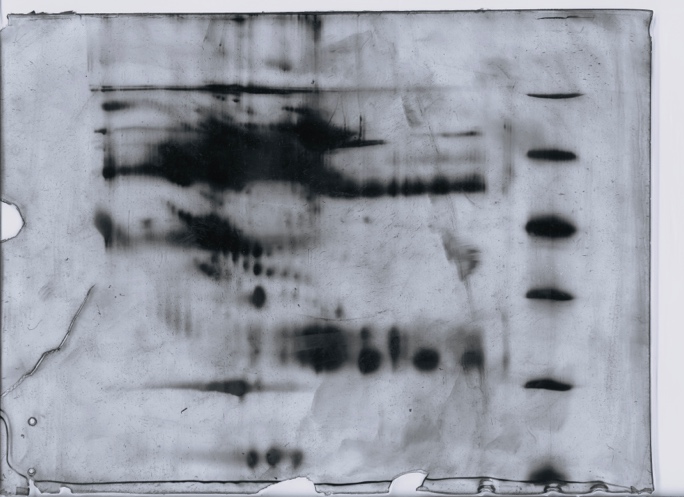


**Supplementary Figure 3.** Determination of differential abundance of proteins in the T2DM+obesity group. Two-dimensional gel electrophoresis of the extracted proteins from newly diagnosed type 2 diabetes mellitus patients with obesity was used to compare protein expression levels against both the control and obesity groups. This figure identifies proteins that may be associated with the progression of obesity to type 2 diabetes mellitus.
